# Editorial: Mesenchymal Stem Cell Senescence and Rejuvenation

**DOI:** 10.3389/fcell.2021.772476

**Published:** 2021-11-25

**Authors:** Yuelin Zhang, Songyan Liao, Qingling Fu, Mark Hamrick

**Affiliations:** ^1^ Department of Emergency Medicine, Guangdong Provincial People’s Hospital, Guangdong Academy of Medical Sciences, Guangzhou, China; ^2^ Department of Medicine, The University of Hong Kong, Hong Kong SAR, China; ^3^ Otorhinolaryngology Hospital, The First Affiliated Hospital of Sun Yat-sen University, Guangzhou, China; ^4^ Medical College of Georgia, Augusta University, Augusta, GA, United States

**Keywords:** mesemchymal stem cells, senescence, rejuvenation, therapy, transplantation

Over the last few decades, stem cell-based therapy has become a novel strategy for a variety of disorders including cardiovascular, digestive and respiratory diseases. Among the stem cells investigated, mesenchymal stem cells (MSCs) are the ideal candidate cell source due to their many characteristics, including ease of isolation and expansion, multilineage differentiation potential and immunoregulatory function. Nevertheless MSCs derived from aged donors or patients with age-related diseases are senescent (Shi et al., 2021). In addition, prolonged *in vitro* expansion induces MSC senescence (Li et al., 2019). Although allogeneic transplantation of MSCs exerts beneficial effects in the early days post-transplantation, long-term benefits compared with those of autologous MSCs are compromised due to sustained immunorejection (Huang et al., 2010). Senescence severely affects the characteristics and function of MSCs, limiting their application in regenerative medicine. Exploring effective strategies to rejuvenate autologous senescent MSC and improve their therapeutic capacity is vital. Although multiple factors including age, oxidative stress and mitochondrial dysfunction have been reported to mediate MSC senescence, the underlying mechanisms of this senescence remain unclear.

There are twelve manuscripts in this Research Topic, highlighting the current understanding of the potential mechanisms underlying MSC senescence and novel strategies to rejuvenate the senescent MSC. Liu et al. and Zhou et al. summarize the current knowledge of phenotypic and biological properties of senescent MSCs and strategies for monitoring and rejuvenation, including gene modification and pretreatment strategies (Liu et al.; Zhou et al.). They also systematically review the molecular mechanisms underlying MSC senescence including epigenetic changes, autophagy, mitochondrial dysfunction and telomere shortening. There is accumulating evidence that autophagy acts as a positive and negative regulator of MSC senescence (Ma et al., 2018; Yang et al., 2018). Rastaldo et al. discuss these conflicting roles of autophagy in MSC senescence and potential mechanistic explanations for such an intricate liaison (Rastaldo et al.). Sirtuin 3 (SIRT3), an NAD + -dependent deacetylase, regulates a variety of physiological and pathological processes including aging and aging-related diseases. Ma et al. have shown that reduced Sirt3 expression contributes to age-related natural senescence and H_2_O_2_-induced premature senescence of rat bone marrow (BM)-MSCs by stimulating cellular reactive oxygen species (ROS) production and DNA injury (Ma et al.). Overexpressing Sirt3 partly reversed the senescence-associated phenotypic features of natural and premature senescent MSCs by alleviating ROS generation and upregulating SOD2 expression. Qin et al. reveal that knockout of NO synthase 2 significantly promoted the adipogenic, but not osteogenic, differentiation capacity of rat MSCs (Qin et al.). Notably, they also showed that knockout of NOS2 in MSCs resulted in significant obesity in rat fed a high-fat diet.

It has been reported that the physiological and pathological condition of the donor plays a critical role in regulating MSC senescence (Alessio et al., 2020; Huang et al., 2019). Yin et al. summarize recent progress in understanding the impact of hyperglycemia on senescence of MSCs and strategies to suppress this senescence in a hyperglycemic environment (Yin et al.). Chen et al. discuss the current knowledge of the role of MSC senescence in the development of myelodysplastic syndromes and show that targeting senescent MSC is a potential strategy for MDS treatment (Chen et al.). Conley et al. report that MSCs isolated from obese subjects display a lower proliferative capacity than those derived from age-matched non-obese subjects. In addition, these obese-MSCs exhibited a senescent phenotype as evidenced by increased p16, p53, IL-6, and MCP-1 gene expression (Conley et al.). Furthermore, co-culture of injured HUVECs with MSCs from non-obese subjects resulted in the formation of tube-like networks but not with MSCs from obese subjects, indicating that pro-angiogenic properties were impaired. Cell source also affects the function of MSCs. Yigitbilek et al. compared the function of liver-derived MSCs (L-MSCs) and adipose tissue-derived MSCs (A-MSCs) from donors matched for gender, age, and body mass index (Yigitbilek et al.). Although both L-MSCs and A-MSCs exhibited a similar senescent phenotype manifested by similar cell cycle arrest and senescence-associated secretory phenotype genes, L-MSCs displayed an enhanced immunomodulatory capacity, while A-MSCs possessed better pro-angiogenic and vascular reparative potency.

Recently, non-coding RNA including lncRNAs and miRNA have been reported to be involved in mediating MSC senescence (Hong et al., 2020; Ren et al., 2021). Dong et al. report that elevated lncRNA lnc-CYP7A1-1 induced human BM-MSC senescence as evidenced by decreased cell proliferative ability, cell survival and migratory ability (Dong et al.). They also found that inhibition of lnc-CYP7A1-1 rejuvenated aged BM-MSCs and improved their therapeutic efficacy in a mouse model of myocardial infarction. There is emerging evidence that MSC-derived exosomes display protective effects against a variety of human diseases (Janockova et al., 2021) although these effects are much reduced in senescent MSCs. Sun. et al. isolated exosomes from young- and aged-MSCs and compared their cardioprotective activities *in vitro* and *in vivo* (Sun et al.). Compared with young-MSC-exosomes, aged-MSC-exosomes exhibited an impaired ability to promote endothelial tube formation and inhibit cardiomyocyte apoptosis *in vitro*. Transplantation of aged-MSC-exosomes also exhibited decreased cardioprotective effects in a mouse model of myocardial infarction. MicroRNA array and PCR analysis revealed dysregulation of miR-221-3p in aged-MSC-exosomes but restoration of miR-221-3p expression rescued aged-MSC-exosome reparative function.

It is well documented that clearance of senescent cells can improve tissue function and extend lifespan during aging (Dookun et al., 2020). To this end, Sharma et al. examined the effects of short-term navitoclax treatment, a chemotherapeutic drug reported to effectively clear senescent cells, on bone mass and osteoprogenitor function in aged mice (Sharma et al.). Interestingly, they found that despite clearance of senescent cells, navitoclax treatment significantly reduced the trabecular bone volume fraction in aged mice and impaired the calcified matrix production by aged BMSC-derived osteoblasts, indicating that the therapeutic effect of navitoclax on age-related bone loss was limited. Future studies including larger-scale studies in rodents and larger animal models are urgently needed to definitively assess the potential therapeutic efficacy of navitoclax in age-related diseases.

In summary, this Research Topic comprises twelve outstanding manuscripts of original research and comprehensive reviews. We summarize the most recent findings regarding the molecular mechanisms underlying MSC senescence and potential strategies for rejuvenation.
